# UVPAR: fast detection of functional shifts in duplicate genes

**DOI:** 10.1186/1471-2105-7-174

**Published:** 2006-03-28

**Authors:** Vicente Arnau, Miguel Gallach, J Ignasi Lucas, Ignacio Marín

**Affiliations:** 1 Departamento de Informática, Universidad de Valencia, Burjassot, Spain; 2 Departamento de Genética, Universidad de Valencia, Burjassot, Spain

## Abstract

**Background:**

The imprint of natural selection on gene sequences is often difficult to detect. A plethora of methods have been devised to detect genetic changes due to selective processes. However, many of those methods depend heavily on underlying assumptions regarding the mode of change of DNA sequences and often require sophisticated mathematical treatments that made them computationally slow. The development of fast and effective methods to detect modifications in the selective constraints of genes is therefore of great interest.

**Results:**

We describe UVPAR, a program designed to quickly test for changes in the functional constraints of duplicate genes. Starting with alignments of the proteins encoded by couples of duplicate genes in two different species, UVPAR detects the regions in which modifications of the functional constraints in the paralogs occurred since both species diverged. Sequences can be analyzed with UVPAR in just a few minutes on a standard PC computer. To demonstrate the power of the program, we first show how the results obtained with UVPAR compare to those based on other approaches, using data for vertebrate *Hox *genes. We then describe a comprehensive study of the RBR family of ubiquitin ligases in which we have performed 529 analyses involving 14 duplicate genes in seven model species. A significant increase in the number of functional shifts was observed for the species *Danio rerio *and for the gene *Ariadne-2*.

**Conclusion:**

These results show that UVPAR can be used to generate sensitive analyses to detect changes in the selection constraints acting on paralogs. The high speed of the program allows its application to genome-scale analyses.

## Background

A major problem in biology is how to convert the data provided by DNA or protein sequences into functional information. For this reason, a significant fraction of molecular evolution studies are focused on the statistical characterization of the patterns of change of DNA or protein sequences. They are based on the general idea that modifications in the function of a gene are often related to changes in the selective regime acting on it, in such a way that a characteristic imprint is generated in its sequence. For example, if a gene is modifying its function by a process that involves positive selection, we would expect to find very rapid changes in the amino acid sequence of the encoded protein, at a rate much higher than expected under neutral evolution. On the contrary, if regions of a gene become functionally constrained, under negative selection, change in those regions will be very slow [1].

Different methods have been proposed to determine how selection acts on biological sequences (reviewed in [2,3]). Several of them have been devised to compare the synonymous (Ks) and non-synonymous (Ka) rates of change in coding regions, often with the purpose of testing whether positive selection has been acting upon those regions [4-6]. However, relative values of Ka/Ks > 1, that are strong evidence for positive selection – being higher than the expected rates under the null hypothesis of neutral evolution (Ka/Ks = 1) – are rarely found. Most frequently, it is determined that sequences are under strong negative selection. For example, in a recent work in which 4706 orthologous genes were compared in human and mouse, it was estimated that, in average, Ka/Ks = 0.107 [7]. Other methods are focused on non-synonymous changes alone, and try to model whether amino acid changes occur homogeneously or, alternatively, are concentrated on particular positions or regions of the proteins [2]. Several other methods have been proposed to explore whether the rates of change of genes vary among different lineages, especially among species or groups of species [8,9]. Specific tools devised to analyze functional divergence between duplicate genes have been also developed [10-13]. This is due to the fact that functional shifts occur especially often in association with gene duplication events. Either by acquisition of new functions by one of the duplicates (neofunctionalization) or by division of the functions of the original gene between the two paralogs (subfunctionalization), duplication is often expected to radically alter the selective forces acting on the duplicate genes [14].

However, most of the approaches developed so far have some serious limitations. First, many of them are computationally cumbersome. Authors often have to choose between using the simplest tools available, that are relatively insensitive, or performing more precise analyses, but with a limited number of genes. A second problem is that many of the methods devised are based on complex mathematical models of how DNA sequences change, and it is often unknown how deviations from the assumptions implicit in those models may affect the conclusions obtained. In fact, there are considerable discussions in the literature on whether certain types of analyses tend to generate spurious significant results [e. g. refs. 15–20]. Therefore, there is a general need for tools that combine the features of being intuitive (i. e. with simple and reasonable underlying assumptions), fast and also sufficiently sensitive.

In this study, we describe a new bioinformatic tool, called UVPAR, which may be used to detect regional changes in constraints in the protein sequences of duplicate genes. The UVPAR algorithm is a substantial refinement of an analytical strategy devised before by one of us and already successfully used for characterizing ancient functional changes in a family of ATPases/ATP synthases [13]. The basis of that strategy is to determine, combining sliding-window analyses of the degree of amino acidic conservation and permutation tests, the regions of duplicate genes that have evolved at different rates in two species.

In its current implementation, UVPAR allows for the fully automatic analysis of a large number of protein sequences in a short period of time. As examples of its potential, we show the results of two analyses. First, we performed UVPAR comparisons for *Hox*7 duplicate genes in six vertebrate species. We demonstrated that some *Hox*7 genes suffered in the past positively selected changes in their sequences associated to functional shifts after gene duplication events [21]. Here, we compare these previous results with the ones obtained with UVPAR in order to establish how our novel method relates to other approaches. As a second example, we generated a comprehensive study of the RBR gene family [22-24] that involves several hundreds of analyses, to show how the program can be used at a large scale. In summary, UVPAR is a useful novel tool for studies focused on the characterization of functional shifts in proteins, most especially in cases in which the genes of interest are part of complex gene families.

## Implementation

**Figure 1:**
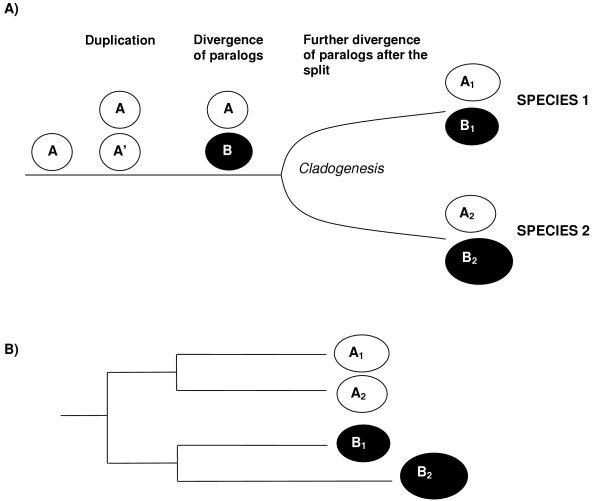
A) Gene duplication followed by divergence of paralogs and the split of two lineages. The gene *A *duplicates to give rise to identical *A *and *A' *paralogs. These paralogs accumulate differences (becoming genes *A *and *B*). Then, the lineages split in such a way that each daughter species conserve the *A *and *B *genes, which can then be called *A_1 _*and *B_1 _*for the first species and *A_2 _*and *B_2 _*for the second species. B) Different rates of evolution, in this case acceleration in the *B_2 _*gene. If this occurs, the genes of the first species (*A_1 _*and *B_1_*) will be more similar than the genes of the second species, a difference that could be detected comparing the sequences with UVPAR.

Let us consider the case in which the phylogenetic analyses of the sequences of certain genes has established that a duplication occurred, generating two paralogous genes, before two lineages of organisms split (Figure 1A, left). After both lineages became separated, the two genes accumulated differences until the present day (Figure 1A, right). The question that we want to tackle is whether sequence changes have accumulated differently in those genes in both lineages since their separation. This often can be visualized as a difference in rates in one or several of the genes in a phylogenetic tree (Figure 1B). UVPAR is a program specialized in analyzing these situations. It takes into account the previous knowledge of orthology/paralogy generated by these phylogenetic analyses. Moreover, UVPAR analyses are based on the multiple-sequence alignments used to generate the phylogenetic trees.

UVPAR is written in C and we have compiled versions for Windows and Linux operating systems. In its simplest implementation, the program uses as input a text file containing the sequences of the proteins encoded by four genes – two duplicate genes in two different species, such as A_1, _B_1_, A_2 _and B_2 _in Figure 1 – in Fasta format. However, the program also accepts larger datasets, in which pairs of duplicates of multiple species are included in a single text file. In this case, the program automatically performs analyses for all the possible combinations of species in the dataset. The program interprets the sequences in the text file as the output of a multiple-sequence alignment (i. e. the amino acid in position *k *in each sequence refers to a characteristic residue, common for all proteins). Once imported, the dataset is filtered, in such a way that positions in which gaps are present are eliminated from all the sequences. The program then detects the sequence with smallest size (*N*) and from then on, all the analyses are performed with the first *N *amino acidic positions of each sequence. After these two filters, UVPAR progressively reads the sequences of the first two proteins (i. e. the duplicates in the first species such as A_1 _and B_1 _in Figure 1) and calculates a value of similarity for each amino acid position of the two sequences, *Bl*_*k1*_, according to a given amino acid substitution matrix. The user may choose among the following matrices: Pam30, Pam70, Pam250, Blosum45, Blosum62 and Blosum90. Blosum62 is the one that we use by default. Then it does the same for the two duplicates of the second species and calculates a similar value, *Bl*_*k2*_. Finally, the program establishes the difference between the two *Bl *values, that we named constraint value, *C(k)_12 _*= *Bl*_*k1 *_- *Bl*_*k2 *_[13]. *C(k)_12 _*is thus a value obtained for "gene quartets", defined as pairs of duplicates in two different species.

If both duplicates are equally different in the two species, the constraint value should be about zero for all the positions. The key of the strategy implemented in UVPAR is to analyze the distribution of *C(k)_12 _*values observed to determine whether some positive or negative values are significantly clustered together. To do so, the vector of *C(k)_12 _*values is randomly shuffled a number *T *of times (according to the algorithm described by Weir [25], p. 386) and the shuffled vectors are compared with the one determined for the real paralogs. The comparison is performed for windows of increasing size (*w*), from *w *= 2 to *w *= *N *- 1. For each window size, the maximum and minimum sums of *C(k)_12 _*values, that we call *S(k, w)*, are determined for both the original and the shuffled sequences. Then, UVPAR compares the maximum and minimum sum in the original sequence with those in the set of shuffled sequences. This set provides a distribution of maximum (or minimum) values against which the values obtained in the real sequence is contrasted. Two hypotheses must be tested for each window size (the first referred to maximum values, the second to minimum values), and therefore it is convenient to use Bonferroni's correction. Thus, when the value in the original sequence is found beyond the top or bottom 2.5 % of the values of the simulated sequences, it is considered significant and UVPAR selects the corresponding window for further analyses.

The second part of the analysis performed by UVPAR is the comparison of all significant windows for a given gene quartet. UVPAR often detects multiple windows of different sizes that are significant. In many cases those windows are nested and therefore they refer in part to the same positions in the analyzed sequences. The biological interpretation of the significant results requires establishing the windows that best explain the detected constraint changes. This problem can be tackled in different ways. In our previous study [13], we solved the problem of nested windows in the simplest way, just choosing the window with the largest (or smallest, for windows of negative sign) value of *S(k, w) *and discarding the rest. This approach was appropriate for the particular cases analyzed in that study, but it has been determined to be insufficient for complex cases found in our subsequent analyses. This is due to two reasons. First, we have found that ties in the *S(k, w) *values often appear in significant windows of different sizes. Second, we have found significant partially overlapping windows, a case that was never detected before. Therefore, in UVPAR we have implemented a much more precise way of dealing with multiple significant windows of different sizes. This is a major improvement on the strategy described on our previously published work

The method is as follows: the program separates the significant windows into two groups, corresponding to those significant in the analyses involving minimum values and those derived from the analyses of maximum values. Within each group, windows are ordered according to their sizes. The program then compares, for one of those two groups, all the significant windows, starting with the smallest one and moving progressively to larger windows. Every time that a window contained in a larger one is found that has an absolute *S(k, w) *value equal or higher than that in the larger window, the latter is eliminated. When this process has finished, the program performs the same search for all the significant windows in the other group. The goal of this iterative screening is to eliminate all the large windows that are significant simply because they contain a small highly deviant region. For example, we found cases in which short regions had such positive *C(k)_12 _*values that even when negative values were added, the larger windows that contains both the highly positive "seed" and the additional negative values were still significantly positive. Those larger windows are eliminated after the comparisons described.

At the end of this first screening, we are left with all the nested significant windows in which an increase of length is associated with an increase of absolute *S(k, w) *value plus all non-nested windows. Then, UVPAR performs a second screening with the remaining windows, this time starting with the largest significant window and comparing it with all the smaller ones. Now, all windows contained in a larger one and at the same time with absolute *S(k, w) *value lower than that found in the larger window are eliminated. The program keeps repeating this search until all windows of both groups (minimum and maximum) have been analyzed. This second screening allows the elimination of all windows that can be extended to larger ones. Logically, the larger windows have to be preferred if their *S(k, w) *values are higher. After these two screenings, we are left with sets of non-nested significant windows, that may or may not partially overlap. As a final step, UVPAR analyzes all the remaining windows and eliminates those with an absolute average *C(k) *value – obtained by dividing the absolute *S(k, w) *value by the window size – lower than 0.1. This last step is devised to eliminate a perverse effect that occurs in rare cases in which a few highly positive or negative *C(k)_12 _*values are clustered together in one of the extremes of the vector. Then, it occasionally occurs that this region not only produces, as expected, a small highly significant window of a particular sign, but also generates a very large complementary window of the opposite sign that often spans the rest of the sequences. This happens even when the *C(k)_12 _*values outside of the short significant region are randomly distributed around zero or, more often, are almost all of them zero. The few cases that we have found in which this effect was present were eliminated when the 0.1 cutoff value was used, and therefore we have considered it to be a logical last filter to be implemented by default in the UVPAR algorithm.

After all the searches are finished, the program generates an output file that details all the relevant parameters: 1) the number of times (*T*) that the sequences have been shuffled to generate the distribution of *S(k, w) *values; 2) the sequences analyzed; 3) the detailed amino acidic alignment, with all the corresponding *C(k)_12 _*values; 4) all the significant windows, before the filtering process; and, 5) all the significant windows, after the filtering process is completed.

Thus, the algorithm can be summarized quite simply as follows:

Import file with the number of pairs of duplicates to be analyzed and corresponding sequences in Fasta format

Read *T *value

Filter sequences to eliminate gaps, estimate final size *N*

Repeat (for each gene quartet)

          Calculate *C(k)_12 _*values

          If (*C(k)_12 _*== 0 for all *k*) then skip gene quartet

Else:

          For (each window size) from *w *= 2 to *w *= *N *-1

          Estimate maximum, minimum *S(k, w) *values for the original sequences

          Shuffle *C(k)_12 _*values, *T *times

          Generate distribution of maximum, minimum *S(k, w) *values from the shuffled sequences

          Compare maximum, minimum *S(k, w) *values for the original and the shuffled sequences, select windows according to a significance level *p *< 2.5 %

          Filter significant windows, increasing size

          Filter significant windows, decreasing size

          Filter significant windows for an average *C(k)_12 _*value > |0.1|

          Generate output file, including: *T *values, sequences, alignments, *C(k)_12 _*values, significant windows before and after filtering

## Results

### Speed and performance

The software that we used in our original study was very slow, what precluded both to use large *T *values and the analysis of large datasets. Due to several highly significant algorithmic improvements, UVPAR is about 10^4 ^times faster. This makes possible to perform the same searches several times and with different *T *values to determine the degree of error associated to each number of permutation tests. When we checked for the impact of *T *values, we determined that to obtain a reasonably precise estimation of the probability associated to a particular value in the distribution of shuffled *S(k, w) *values, it is convenient to choose values of *T *larger than 10^3^, which we previously used due to computational limitations. We have fully repeated the analyses presented in [13] using UVPAR and, although all the conclusions of the work are correct, we have found that the error associated to the estimation of p values with *T *= 10^3 ^was considerable (about ± 0.5 %). UVPAR analyses are fast enough as to easily handle values of *T *= 10^4^, which we consider now to be the minimum number of permutation tests to be used in this type of studies. If the sequences are short enough,*T *may be increased to 10^5 ^or even 10^6^. As examples, using *T *= 10^4 ^and a standard PC computer (Intel Pentium IV 2.8 GHz processor with 1 GB RAM memory), sequence quartets of 150 amino acids can be analyzed in about 16 seconds, quartets of 500 amino acids in about 6 minutes and quartets of long sequences of 1000 amino acids in about 26 minutes. Time only vary slightly depending on the number of significant windows found. These results mean that UVPAR can be used at a large scale. As an example, the analysis of a whole family of proteins that we will detail in section 4.3, that involved more than 500 individual UVPAR searches (protein length = 142 amino acids, *T *= 10^5^) required a computation time of only 22 hours, about 2.5 minutes per analysis.

### Simulations to determine the reliability and sensitivity of UVPAR searches

**Figure 2:**
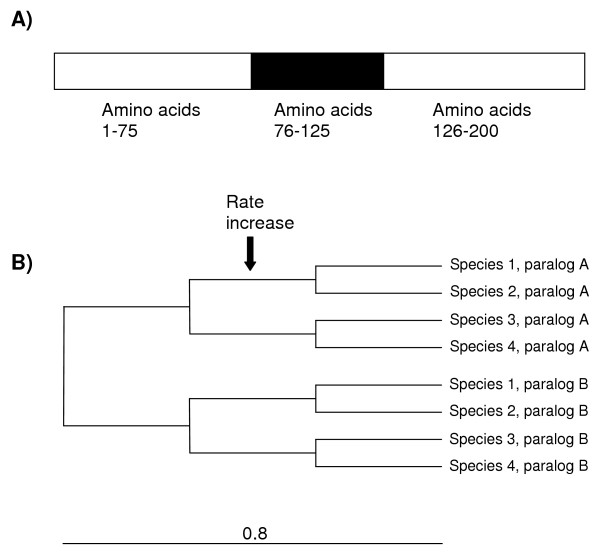
A) Structure of the simulated proteins. Total length was 200 amino acids. The region in which different evolutionary rates were tested correspond to positions 76–125. B) Topology of the tree in which the simulations were performed. Total branch length is 0.8 and the length of all inner branches is 0.8/3 = 0.267. The default average substitution rate was set to μ = 0.5. This rate was increased to μ = 1, μ = 2.5 or μ = 5 in the 76–125 region to test for the effect of local rate increase on UVPAR results. The α parameter of the gamma distribution of rate heterogeneity among sites was varied between 0.2 and 3 (see text and Table 1). A total of 100 simulations were performed for each combination of μ and α.

To check for the number of false positives and false negative results generated by UVPAR under different conditions, we explored, using simulations, a tree containing four species, with two paralogs each (details in Figure 2). We used CovTree [26] to apply different changes in the rates of a particular region of one of the paralogs in the branch previous to the split between two of the species (species 1 and 2 in Figure 2). Then, we used UVPAR to search for coherent significant windows, i. e. windows that included at least part of the region in which the rate shifts were applied and that were present in all the four comparisons in which we would expect to find significant results after the rate shifts (species 1 vs. species 3; species 1 vs. species 4; species 2 vs. species 3; and species 2 vs. species 4). Simulations were performed using sequences with different degrees of heterogeneity of evolutionary rates among sites. Heterogeneity was modeled as a gamma distribution with a variable α parameter. Values of α from 0.2 (highly heterogeneous rates among amino acids) to α = 3 (highly homogeneous rates) were used, to cover the range of results detected for real proteins [ref. [27]; the average α detected in that study was 0.71]. Results are shown in Table 1 for windows of size *w *= 5 (i. e. at least 10% of the size of the modified region; these are about 95% of the significant windows found in the simulations). We found only 2 – 3% of cases in which coherent windows are present in the control simulations (in which no rate shift was applied) no matter the value of α. When the rate of evolution for the region of interest is increased, more cases are detected, and an increase in the average *C(k)_12 _*is also observed. If rates are increased ten times and α values are relatively large (α ≥ 0.7), coherent windows are detected in 35% to 55% of the cases (Table 1). Negative results in these simulations are caused by random changes that obscure the effect of the rate increase. Very low α values (α = 0.2) in general precluded the detection of regional shifts by UVPAR. These results suggest that, in cases in which multiple sequences can be analyzed, systematic positive UVPAR results most likely will correspond to real regional changes. They also indicate that UVPAR losses sensitivity when α values are very small and that the program does not detect small shifts in evolutionary rates; changes must be strong to generate a significant signal for UVPAR analyses.

### Functional shifts in Hox7 genes

*Hox *genes are critical in many basic developmental processes, most especially in the determination of cell fates along the anterior-posterior body axis [28,29]. Vertebrates have a large number of *Hox *genes, generated by gene and genome duplications [30-32]. In a previous work, we showed that episodes of positive selection affected *Hox7 *genes in vertebrate species [21]. More precisely, we determined that both the ancestral duplication that gave rise to the paralogous *Hox-a7 *and *Hox-b7 *genes and the tetraploidization that occurred in the amphibian lineage that includes the model species *Xenopus laevis *radically altered the selective regime acting on those genes. In the first case, the duplication was followed by a period of time in which *Hox-a7 *genes diversified under positive selection. In the second case, one of the *Hox-b7 *genes that were produced in the *Xenopus *ancestor by the genome duplication process (called *Hox-b7b*) also changed under positive selection. The ratios of non-synonymous vs. synonymous substitutions in the branches affected by those two selective shifts were estimated to be about eight to ten times larger that the average ratio for the rest of branches of the tree [21]. Three distinct types of analyses suggested that positive selection acted on the regulatory N-terminal region of the HOX7 proteins, while the highly conserved homeodomain was not affected [21].

**Figure 3:**
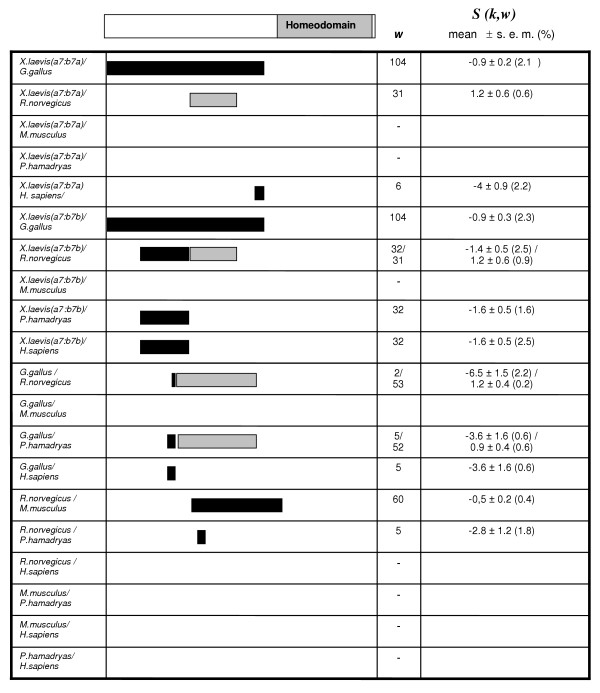
Distribution of significant results in the 20 *Hox-a7-Hox-b7 *comparisons. Grey: significant positive *S(k, w) *values (the duplicates of the first named species are the most conserved), Black: significant negative *S(k, w) *values (the second species has the most conserved duplicates). *w*: window size.

The large shift in evolutionary rates and the relative high values of α for these sequences (α = 0.65 for the whole sequences, and α = 1.92 for the N-terminal region; data calculated according to [33]) mean that this is a favorable case to test whether UVPAR is able to generate results comparable to those found with more complex methods. Figure 3 shows the results for UVPAR analyses (*T *= 10^5^) for the six vertebrates in which both duplicates, *Hox-a7 *and *Hox-b7*, were analyzed in our previous work (*Xenopus laevis*, *Gallus gallus*, *Rattus norvegicus*, *Mus musculus*, *Papio hamadryas *and *Homo sapiens*). Alignments were the same used in [21]. UVPAR results are congruent with two of the main conclusions of our previous work. First, comparisons involving the *Xenopus Hoxb7-b *gene showed significant shifts when compared with *Hox7 *genes of other vertebrates in 4 out of 5 comparisons (Figure 3). The exception, the comparison involving *Mus musculus*, is mainly due to a few homoplastic residues in the *Mus musculus Hoxb7 *sequence which preclude the overall values to become significant. Second, all significant windows were found outside of the homeodomain (see scheme for the proteins in Figure 3, top). It must be noted that the third main result – the rapid differentiation after the *Hox-a7*/*Hox-b7 *duplication – could not be possibly detected here because that process occurred before all lineages that can be analyzed with UVPAR became separate. In summary, these UVPAR results agree with our previous findings and show that UVPAR is able to provide sensitive evidence for functional shifts comparable to those obtained by more complex and time-consuming mathematical approaches.

### UVPAR analysis of RBR ubiquitin ligases

We decided to demonstrate the ability of the program to handle large datasets by analyzing proteins of a family, called RBR, that we defined some time ago [22]. RBR proteins are ubiquitin ligases with important roles in the control of protein degradation and several of them are known to be involved in human diseases (reviewed in [23]). RBR proteins are characterized by having the RBR supradomain, that is composed by two RING fingers (RING1 and RING2; although RING2 is actually quite different from a canonical RING finger, see [34]) that are separated by an IBR domain [35].

**Figure 4:**
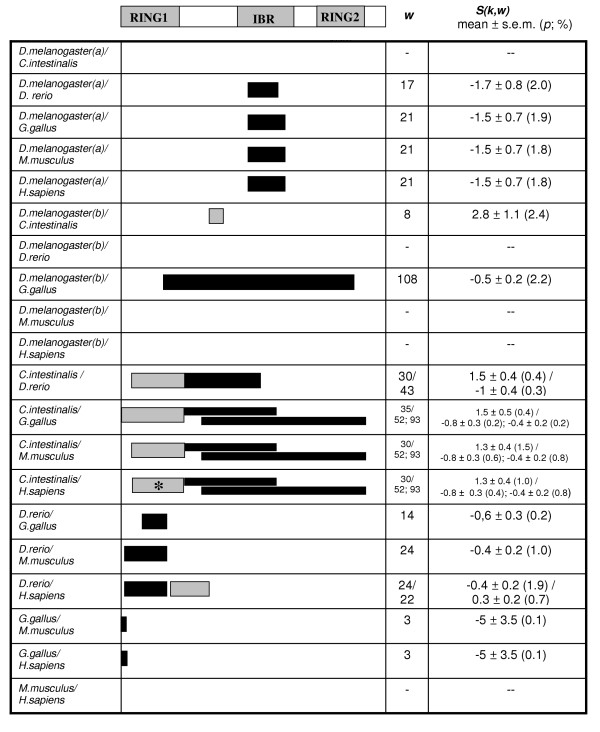
Significant results in the 20 comparisons involving the *ARI1 *and *ARI2 *genes. Data shown as in Figure 1. (a): comparisons involving *Drosophila ari-1a*; (b): comparisons with *Drosophila ari-1b*. The asterisk refers to the region shown in Figure 4.

We checked for modifications in the functional constraints by analyzing the RBR supradomain of multiple RBR proteins in several model organisms. We took advantage of the recent completion by our group of a comprehensive analysis in which we generated protein alignments for 347 RBR proteins and phylogenetic trees that allowed us to determine the orthology/paralogy relationships for all members of the family [24]. From that analysis, we selected all the available sequences for 14 RBR genes (*ARI1*, *ARI2*, *ANKIB1*, *Parc*, *Paul*, *IBRDC1*, *Parkin*, *XAP3*, *RNF144*, *IBRDC2*, *Dorfin*, *IBRDC3*, *ARA54 *and *Triad3*, according to their human nomenclature) in seven model organisms (*Caenorhabditis elegans*, *Drosophila melanogaster*, *Ciona intestinalis*, *Danio rerio*, *Gallus gallus*, *Mus musculus *and *Homo sapiens*). The estimated average value of α for these sequences is 1.08. Therefore sites evolve homogeneously enough for UVPAR analyses. We took into account that not all the genes are present in all the species analyzed (e. g. some are vertebrate-specific), and some of the genes had species-specific duplications. Once these peculiarities were sorted out, we performed all the possible combinations of gene quartets for the seven species. A total of 529 analyses were generated. In 138 cases (26.1 %), we found significant results. They are summarized in Tables 2 and 3, according to, respectively, the species and genes involved (see details in [Additional file 1]). Table 2 shows that a single species, the fish *Danio rerio*, present a number of significant results that are well above the average found for the set of species. We think that, similarly to what occurred for the *Xenopus Hox7 *genes shown above, this may be related to the genome duplication that happened in the evolutionary lineage in which *Danio *emerged [36]. If we compare the data for the different RBR genes (Table 3), we observe that the significant cases appear quite homogeneously. *ARI2 *is the only gene with a number of significant results that is in average higher than that of the rest of RBRs. This is in part explained by the large number of analyses (15 out of 20) in which the *ARI1*/*ARI2 *comparisons were significant. Figure 4 shows the results for those comparisons. It becomes evident examining this figure that many of the positive results are correlated. In *Drosophila melanogaster *there are two *ARI1 *genes, *ari-1a *and *ari-1b *(the slightly different gene nomenclature is the official one for *Drosophila *genes). Significant shifts in the IBR domain are observed between the pair *ari-1a*/*ari-2 *of *Drosophila melanogaster *and the *ARI1*/*ARI2 *genes of all vertebrate species. Also, we see important changes along the whole RBR supradomain when genes of the urochordate *Ciona intestinalis *are compared with vertebrate genes. Therefore all these results can be explained by a few major changes in particular evolutionary lineages. In favorable cases, we can establish in which lineage the changes must have occurred. For example, considering that *D. melanogaster*/vertebrate comparisons involving the *D. melanogaster ari-1b *gene are in general non-significant, we can conclude that a functional shift in the IBR domain of the *D. melanogaster ari-1a *gene occurred since the *ari1-a/ari1-b *duplication that has made *ari-1a *different from their vertebrate orthologs. In summary, the results shown in Figure 4 suggests that functional diversification between the RBR supradomains of the *ARI1 *and *ARI2 *genes has occurred just a few times and that those changes most frequently affected the RING1 and IBR domains. This result points towards an important role of the N-terminal domains of the RBR supradomain in acquisition of novel functional properties in some species, perhaps to act on new ubiquitination substrates, while the non-canonical RING2 would be less significant.

**Figure 5:**
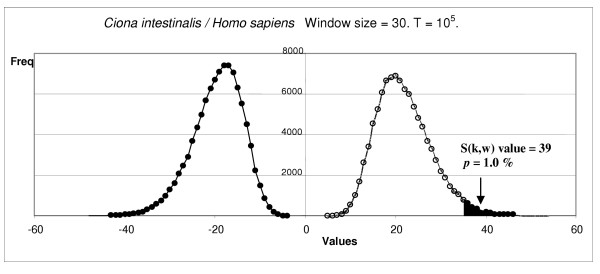
Distribution of maximum and minimum values of the shuffled sequences for one of the cases shown in Figure 3: RING1 shift between *Ciona intestinalis *and *Homo sapiens*. The arrow shows the value observed for the significant window.

In Figure 5, we show the distribution of maximum and minimum values in the shuffled sequences and the relative position in this distribution for one of the significant values observed. Bell shaped, quasi-symmetrical distributions as those shown in that figure are common. They correspond to the typical extreme value distributions that are expected for large *T *and *w *values ([37], p. 151). We have however observed in some cases highly asymmetrical distributions, often with multiple peaks, when window sizes are small.

## Discussion

We have shown that the UVPAR program to may be used to quickly detect modifications in the functional constraints of duplicate genes. Favorable circumstances for the program are a reasonably homogenous rate of amino acid substitution among sites and an intense change in the selective regime acting on one of the paralogs. Considering that under positive selection Ka/Ks > 1 while in real proteins, the average value is much lower (e. g. Ka/Ks = 0.107 for the large sample of human/mouse comparisons already mentioned [7]), we may expect UVPAR to be particularly useful to detect positive selection, most especially associated to functional shifts after gene duplication. In respect to other related methods, that often rely on debatable *a priori *assumptions and difficult mathematical calculations, UVPAR results are easy to obtain and highly intuitive. UVPAR analyses depend on just two *a priori *suppositions. First, that protein changes can be measured with a matrix such as Blosum62. Second, that the shifts affect contiguous amino acids, i. e. regions of the proteins, and therefore they can be detected by examining their primary sequences, using a sliding-window approach. There is strong evidence that regional shifts in proteins sequences often occur, in genome-scale analyses [38]. UVPAR advantages are most obvious when the goal is to quickly explore, across many species, complex gene families that include several duplicate genes. This is clearly shown by the analysis of the RBR family described above. We have also demonstrated that general conclusions about what genes or species are more prone to suffer functional shifts can be deduced from the comprehensive study of gene families (Tables 2 and 3). The fact that many genes can be examined in a very short time opens up the possibility of performing genome-scale studies, impossible to generate with any other related method.

An obvious statistical problem is that, when a large number of quartets are examined with UVPAR tests, the quite permissive significance level used (5%) is expected to generate a number of false positive results. Our experience, as well as the simulations shown above, suggests examining three aspects of the data to obtain conclusions not affected by that problem. These three aspect are: 1) The significance level of each positive result and the average value of *C(k)_12 _*for the significant windows. In our simulations, false positive windows have low average *C(k)_12 _*values (Table 1). If it is deemed necessary, the analyses can be made more strict by either changing the significance level or by increasing the conventional cutoff value *C(k)_12 _*> |0.1|; 2) The logic in structural or functional terms of the results obtained (e. g. to observe whether they refer to known domains of a protein, regions in close proximity in its three-dimensional structure, etc.); and, 3) The systematic consistency of the results for multiple species. In our opinion, internal consistency of the data is a particularly useful way to filter the data for real positives. This is clearly observed in our simulations, in which we checked for the consistency of UVPAR results for multiple analyses, finding that false positive coherent results are rare (2 – 3% under the conditions tested). Thus, when it is found, as we have shown for the *ARI1*/*ARI2 *data (Figure 4), that the genes of multiple species of a lineage (e. g. vertebrates such as fishes, birds and mammals) generate the same results when compared with genes of an outgroup species (*Drosophila*, *Ciona*), it is good evidence for a real shift to have occurred. This means that UVPAR finds several times the same imprint even although the analyzed sequences are considerably distinct. After all, orthologous genes of fishes, birds and mammals are separated by hundreds of millions of years of independent evolution.

The combination of UVPAR results for multiple species may be often used to trace with precision the moment and the lineage in which a change in functional constraints occurred. In this study, we have shown that the duplication of *Hox*7 genes in *Xenopus laevis *and of *ari-1 *genes in *Drosophila melanogaster *were associated with modifications in the constraints of at least one of the duplicates. These conclusions can be easily deduced when the data are presented in a favorable format, as that shown in Figures 3 and 4. Our approach may thus complement other analytical methods devised to detect selective processes acting on particular branches of a phylogenetic tree [8,9,21].

## Conclusion

UVPAR generates very fast and still sensitive information about modifications of the selective constraints in duplicate genes. It can be used at a large scale, to analyze complex gene families in multiple species. Its speed, simplicity of use and also the fact that its mathematical assumptions are very intuitive makes UVPAR an excellent tool for all groups interested in analyzing the impact of natural selection on gene sequences, especially at a genomic scale.

## Availability

UVPAR is written in C. Windows and Linux versions of UVPAR are available, free for academic users, at the following web page: . In that page, an example of how to use UVPAR can also be found. Non-academic users may obtain a license to use or distribute the program by contacting the corresponding author.

## Authors' contributions

VA wrote the UVPAR code and contributed to the improvements in the analytical strategy implemented in the program. MG and IL performed the *Hox *and RBR analyses as well as the simulations presented above. MG also contributed to the development of the analytical strategy and tested different versions of the program. The basic strategy implemented in UVPAR was developed by IM, who also coordinated the research, contributed to the improvements in the strategy shown here and wrote the manuscript. All authors read and approved the final manuscript.

## Supplementary Material

Additional File 1 Table 1: Significant regions for comparisons among RBR genes. Click here for file

## Figures and Tables

**Table 1:** Results of the simulations: Percentage of quartets in which significant windows were found and *C(k)_12 _*values (average ± s.e.m.) for those windows. The values of μ refer to the rate applied to the region shown in Figure 1 (amino acids 76 – 125 in the simulated proteins), while the other regions of the sequences (amino acids 1 – 75 and 126 – 200) were kept evolving at a basal rate μ = 0.5. α is the shape parameter of the gamma distribution. The percentage of significant simulations was compared between the controls and the quartets with increased rates using the chi-square test. The average *C(k)*_*12 *_values were compared using the t test. In bold, significant results. p values are detailed

	**α = 0.2**	**α = 0.7**	**α = 1.5**	**α = 3**
**μ = 0.5 ** (control, no increase)	2% 0.881 ± 0.146	3% 0.664 ± 0.208	2% 0.715 ± 0.238	3% 0.831 ± 0.226
**μ = 1 ** (2× increase)	2% 0.613 ± 0.306	3% 0.583 ± 0.218	3% 0.914 ± 0.124	3% 0.909 ± 0.102
**μ = 2.5 ** (5× increase)	5% 0.814 ± 0.133	**13% (p = 0.012) ** **0.998 ± 0.070 (p = 0.037)**	**12% (p = 0.008)** 0.808 ± 0.075	**14% (p = 0.007)** 1.010 ± 0.096
**μ = 5 ** (10× increase)	**10% (p = 0.020)** 1.048 ± 0.220	**35% (p < 0.001)** **1.188 ± 0.045** **(p = 0.002)**	**55% (p < 0.001)** **1.328 ± 0.114** **(p = 0.001)**	**51% (p < 0.001)** **1.374 ± 0.049** **(p = 0.006)**

**Table 2:** Summary of significant results for comparisons of RBR proteins, according to the examined species. The table shows the number to significant results (one or more significant windows) divided by the total number of comparisons for each pair of species. Significance levels (*p*) to reject the null hypothesis of identical number of positive results among species were determined using a cumulative hypergeometric distribution taking into account the global fraction of positives (138/529). ns: not significant after Bonferroni's correction (i. e.*p *> 0.05/7)

	*C. elegans*	*D. melanogaster*	*C. intestinalis*	*D. rerio*	*G. gallus*	*M. musculus*	*H. sapiens*
*C. elegans*	--	0/1	0/1	0/2	4/9	3/9	1/9
*D. melanogaster*	0/1	--	1/11	3/11	8/11	3/17	5/17
*C. intestinalis*	0/1	1/11	--	6/16	7/13	6/25	7/25
*D. rerio*	0/2	3/11	6/16	--	10/27	23/62	21/62
*G. gallus*	4/9	8/11	7/13	10/27	--	8/55	12/55
*M. musculus*	3/9	3/17	6/25	23/62	8/55	--	10/91
*H. sapiens*	1/9	5/17	7/25	21/62	12/55	10/91	--
TOTAL	8/31	20/68	27/91	63/180	49/170	53/259	56/259
%	25.8	29.4	29.7	35.0	28.8	20.5	21.6
*p*	ns	ns	ns	**0.0007**	ns	ns	ns

**Table 3:** Summary of significant results, classified according to the genes examined. The fractions refer to number of significant results/total number of comparisons for each pair of orthologous genes. The hypothesis of identical number of positives among genes was tested as described in Table 1. ns: non-significant after Bonferroni's correction (*p *> 0.05/14)

	*ARI1*	*ARI2*	*ANKIB1*	*PARC*	*PAUL*	*IBRDC1*	*PARKIN*	*XAP3*	*RNF144*	*IBRDC2*	*DORFIN*	*IBRDC3*	*ARA54*	*TRIAD3*
*ARI1*	--	15/20	0/6	4/6	0/14	0/3	2/9	4/9	13/20	0/6	0/3	0/6	0/6	2/6
*ARI2*	15/20	--	4/6	5/6	0/10	2/3	4/10	5/9	3/15	4/15	1/10	0/10	1/10	3/6
*ANKIB1*	0/6	4/6	--	0/3	2/3	0/3	1/3	0/5	3/6	4/6	0/6	0/6	0/3	3/6
*PARC*	4/6	5/6	0/3	--	0/3	0/1	2/3	2/3	0/6	1/6	0/3	0/3	2/6	0/3
*PAUL*	0/14	0/10	2/3	0/3	--	2/3	0/3	6/9	0/10	1/10	0/3	0/3	0/3	2/3
*IBRDC1*	0/3	2/3	0/3	0/1	2/3	--	0/1	0/5	1/3	0/3	2/3	0/3	0/1	0/3
*PARKIN*	2/9	4/10	1/3	2/3	0/3	0/1	--	0/1	2/6	1/6	2/6	2/6	1/6	0/3
*XAP3*	4/9	5/9	0/5	2/3	6/9	0/5	0/1	--	3/9	0/5	0/1	2/5	0/3	3/5
*RNF144*	13/20	3/15	3/6	0/6	0/10	1/3	2/6	3/9	--	0/6	0/6	1/6	3/6	0/6
*IBRDC2*	0/6	4/15	4/6	1/6	1/10	0/3	1/6	0/5	0/6	--	1/6	1/6	1/6	4/6
*DORFIN*	0/3	1/10	0/6	0/3	0/3	2/3	2/6	0/1	0/6	1/6	--	3/6	0/6	0/6
*IBRDC3*	0/6	0/10	0/6	0/3	0/3	0/3	2/6	2/5	1/6	1/6	3/6	--	1/6	1/6
*ARA54*	0/6	1/10	0/3	2/6	0/3	0/1	1/6	0/3	3/6	1/6	0/6	1/6	--	0/3
*TRIAD3*	2/6	3/6	3/6	0/3	2/3	0/3	0/3	3/5	0/6	4/6	0/6	1/6	0/3	--
TOTAL	40/114	47/130	17/62	16/52	13/77	7/35	17/63	25/69	29/105	18/87	9/65	11/72	9/65	18/62
%	35.1	36.2	27.4	30.8	16.9	20.0	27.0	36.2	27.6	20.7	13.8	15.3	13.8	29.0
***p***	ns	**0.0022 **	ns	ns	ns	ns	ns	ns	ns	ns	ns	ns	ns	ns
