# Supplementary material for: UVPAR: fast detection of functional shifts in duplicate genes
Source: BMC Bioinformatics. 2006 Mar 28;7:174. doi: 10.1186/1471-2105-7-174 (PMC1570150; doi:10.1186/1471-2105-7-174)
Supplement: Additional File 1 — Table 1: Significant regions for comparisons among RBR genes. [file 1471-2105-7-174-S1.doc]

**Supplementary Table 1.** Significant regions for comparisons among RBR genes. We indicate the size of the significant window, its position in the alignment and the average values and standard errors of the mean for *S(k,w)*. The percentage of windows with values more extreme that those shown are also indicated.

| **Genes, pairs of species** | **size** | **position** | **mean ± s.e.m**  **of S(k,w) (p; %)** |
| --- | --- | --- | --- |
|  |  |  |  |
| ***ARI1_ARI2*** |  |  |  |
| *D. rerio/D. melanogaster (a)* | 17 | 72-88 | 1.65±0.80 (1.93) |
| *G. gallus/D. melanogaster (a)* | 21 | 72-92 | 1.48±0.65 (1.89) |
| *M. musculus/D. melanogaster (a)* | 21 | 72-92 | 1.52±0.65 (1.76) |
| *H. sapiens/D. melanogaster (a)* | 21 | 72-92 | 1.52±0.65 (1.78) |
| *C. intestinalis/D. melanogaster (b)* | 8 | 50-57 | -2.75±1.08 (2.42) |
| *G. gallus/Dr. melanogaster (b)* | 108 | 24-131 | 0.50±0.23 (2.23) |
| *D. rerio/C. intestinalis* | 30 | 6-35 | -1.53±0.43 (0.38) |
| *D. rerio/C. intestinalis* | 43 | 36-78 | 0.98±0.41 (0.25) |
| *G. gallus/C. intestinalis* | 35 | 1-35 | -1.51±0.46 (0.36) |
| *G. gallus/C. intestinalis* | 52 | 36-87 | 0.83±0.34 (0.25) |
| *G. gallus/C. intestinalis* | 93 | 39-131 | 0.41±0.22 (0.19) |
| *M. musculus/C.intestinalis* | 30 | 6-35 | -1.27±0.40 (1.44) |
| *M. musculus/C.intestinalis* | 52 | 36-87 | 0.79±0.33 (0.61) |
| *M. musculus/C.intestinalis* | 93 | 39-131 | 0.39±0.22 (1.11) |
| *H. sapiens/C. intestinalis* | 30 | 6-35 | -1.30±0.40 (0.10) |
| *H. sapiens/C. intestinalis* | 52 | 36-87 | 0.83±0.33 (0.35) |
| *H. sapiens/C. intestinalis* | 93 | 39-131 | 0.41±0.21 (0.71) |
| *G. gallus/D. rerio* | 14 | 12-25 | 0.64±0.25 (0.22) |
| *M. musculus/D. rerio* | 24 | 2-25 | 0.42±0.16 (0.98) |
| *H. sapiens/D. rerio* | 24 | 2-25 | 0.42±0.16 (1.85) |
| *H. sapiens/D. rerio* | 22 | 28-49 | -0.32±0.18 (0.67) |
| *M. musculus/G. gallus* | 3 | 1-3 | 5.00±3.51 (0.13) |
| *H. sapiens/G. gallus* | 3 | 1-3 | 5.00±3.51 (0.12) |
|  |  |  |  |
| ***ARI1_PARC*** |  |  |  |
| *G. gallus/C. intestinalis* | 116 | 16-131 | 0.50±0.24 (0.31) |
| *M. musculus/C.intestinalis* | 87 | 16-102 | 0.47±0.26 (1.09) |
| *M. musculus/C.intestinalis* | 109 | 18-126 | 0.32±0.22 (0.78) |
| *H. sapiens/C. intestinalis* | 87 | 16-102 | 0.48±0.27 (2.46) |
| *H. sapiens/C. intestinalis* | 110 | 18-127 | 0.35±0.23 (0.98) |
| *M. musculus/G. gallus* | 13 | 76-88 | 1.08±0.60 (1.23) |
|  |  |  |  |
| ***ARI1_PARKIN*** |  |  |  |
| *G. gallus/D. melanogaster* | 6 | 115-120 | 3.17±1.35 (1.54) |
| *H. sapiens/D. melanogaster* | 6 | 115-120 | 3.33±1.36 (1.82) |
|  |  |  |  |
| ***ARI1_XAP3*** |  |  |  |
| *D. rerio (a)/C. intestinalis* | 109 | 24-132 | 0.22±0.16 (2.30) |
| *D. rerio (a)/C. intestinalis* | 2 | 140-141 | -8.00±0.00 (1.40) |
| *M. musculus/D. rerio (a)* | 108 | 24-131 | -0.26±0.12 (0.15) |
| *M. musculus/D. rerio (a)* | 104 | 30-133 | -0.25±0.14 (2.48) |
| *M. musculus/D. rerio (a)* | 104 | 31-134 | -0.25±0.14 (2.48) |
| *M. musculus/D. rerio (a)* | 104 | 32-135 | -0.25±0.14 (2.48) |
| *M. musculus/D. rerio (a)* | 2 | 140-141 | 8.00±0.00 (1.40) |
| *H. sapiens/D. rerio (a)* | 100 | 32-131 | -0.34±0.12 (0.29) |
| *H. sapiens/D. rerio (a)* | 2 | 140-141 | 8.00±0.00 (1.35) |
| *H. sapiens/M. musculus* | 43 | 45-87 | -0.28±0.15 (1.23) |
|  |  |  |  |
| ***ARI1_RNF144*** |  |  |  |
| *D. rerio/D. melanogaster (a)* | 70 | 44-113 | 0.80±0.34 (0.69) |
| *G. gallus/D. melanogaster (a)* | 95 | 19-113 | 0.57±0.30 (0.98) |
| *M. musculus/D. melanogaster (a)* | 95 | 19-113 | 0.48±0.29 (1.46) |
| *H. sapiens/D. melanogaster (a)* | 95 | 19-113 | 0.58±0.29 (0.84) |
| *D. rerio/D. melanogaster (b)* | 92 | 24-115 | 0.63±0.26 (1.97) |
| *D. rerio/D. melanogaster (b)* | 95 | 19-113 | 0.6±0.255 (2.42) |
| *G. gallus/D. melanogaster (b)* | 97 | 19-115 | 0.63±0.25 (2.00) |
| *H. sapiens/D. melanogaster (b)* | 97 | 19-115 | 0.64±0.24 (1.52) |
| *D. rerio/C. intestinalis* | 70 | 62-131 | 0.87±0.30 (1.50) |
| *G. gallus/C. intestinalis* | 119 | 13-131 | 0.52±0.21 (2.49) |
| *H. sapiens/C. intestinalis* | 118 | 14-131 | 0.53±0.21 (2.46) |
| *M. musculus/D. rerio* | 28 | 77-104 | -0.82±0.46 (1.05) |
| *H. sapiens/D. rerio* | 6 | 99-104 | -2.17±1.30 (0.92) |
| *H. sapiens/G. gallus* | 9 | 95-103 | -1.22±0.60 (0.97) |
|  |  |  |  |
| ***ARI1_TRIAD3*** |  |  |  |
| *M. musculus/D. rerio* | 137 | 1-137 | 0.13±0.10 (2.15) |
| *M. musculus/D. rerio* | 137 | 2-138 | 0.13±0.10 (2.15) |
| *H. sapiens/D. rerio* | 138 | 1-138 | 0.11±0.10 (1.63) |
| *H. sapiens/D. rerio* | 138 | 2-139 | 0.11±0.10 (1.63) |
|  |  |  |  |
| ***ARI2_ANKIB1*** |  |  |  |
| *G. gallus/D. rerio* | 3 | 1-3 | -7.67±2.96 (0.07) |
| *G. gallus/D. rerio* | 82 | 15-96 | 0.28±0.10 (1.66) |
| *M. musculus/D. rerio* | 28 | 59-86 | 0.71±0.25 (1.54) |
| *H. sapiens/D. rerio* | 37 | 50-86 | 0.62±0.20 (2.10) |
| *M. musculus/G. gallus* | 30 | 32-61 | -0.30±0.17 (2.19) |
|  |  |  |  |
| ***ARI2_PARC*** |  |  |  |
| *G. gallus/C. intestinalis* | 115 | 12-126 | 0.59±0.20 (0.28) |
| *M. musculus/C.intestinalis* | 17 | 47-63 | 2.12±0.86 (2.16) |
| *H. sapiens/C. intestinalis* | 14 | 47-60 | 2.50±0.84 (1.64) |
| *H. sapiens/G. gallus* | 32 | 79-110 | -0.44±0.17 (1.88) |
| *H. sapiens/G. gallus* | 32 | 1-32 | 1.00±0.46 (1.87) |
| *H. sapiens/M. musculus* | 37 | 6-42 | 0.32±0.18 (0.93) |
|  |  |  |  |
| ***ARI2_IBRDC1*** |  |  |  |
| *M. musculus/D. rerio* | 70 | 36-105 | -0.37±0.12 (1.07) |
| *H. sapiens/D. rerio* | 70 | 36-105 | -0.36±0.12 (0.64) |
|  |  |  |  |
| ***ARI2_PARKIN*** |  |  |  |
| *G. gallus/C. elegans* | 139 | 4-142 | 0.71±0.26 (2.28) |
| *G. gallus/C. elegans* | 140 | 2-141 | 0.7±0.26 (2.18) |
| *G. gallus/D. melanogaster* | 134 | 2-135 | 0.49±0.23 (2.20) |
| *M. musculus/D. melanogaster* | 10 | 50-59 | -2.80±1.23 (0.37) |
| *H. sapiens/D. melanogaster* | 10 | 50-59 | -2.70±1.18 (0.47) |
|  |  |  |  |
| ***ARI2_XAP3*** |  |  |  |
| *D. rerio (a)/C. intestinalis* | 18 | 34-51 | 1.83±0.58 (2.21) |
| *M. musculus/C.intestinalis* | 9 | 43-51 | 2.56±0.80 (2.39) |
| *H. sapiens/C. intestinalis* | 9 | 43-51 | 2.67±0.76 (2.25) |
| *M. musculus/D. rerio (a)* | 139 | 1-139 | -0.14±0.12 (1.41) |
| *H. sapiens/D. rerio (a)* | 139 | 1-139 | -0.14±0.13 (1.70) |

| **ARI2_RNF144** |  |  |  |
| --- | --- | --- | --- |

| *G. gallus/D. melanogaster* | 136 | 3-138 | 0.51±0.20 (2.38) |
| --- | --- | --- | --- |
| *G. gallus/C. intestinalis* | 127 | 15-141 | 0.48±0.23 (0.97) |
| *G. gallus/C. intestinalis* | 124 | 19-142 | 0.48±0.23 (2.38) |
| *M. musculus/G. gallus* | 52 | 35-86 | -0.33±0.23 (0.87) |
|  |  |  |  |
| ***ARI2_IBRDC2*** |  |  |  |
| *G. gallus/D. melanogaster* | 139 | 3-141 | 0.19±0.18 (2.28) |
| *G. gallus/C. intestinalis* | 12 | 1-12 | -2.75±1.12 (2.44) |
| *G. gallus/C. intestinalis* | 127 | 15-141 | 0.20±0.21 (0.71) |
| *G. gallus/D. rerio* | 135 | 7-141 | 0.13±0.12 (1.96) |
| *G. gallus/D. rerio* | 135 | 8-142 | 0.13±0.12 (1.96) |
| *M. musculus/D. rerio* | 3 | 86-88 | -3.67±1.20 (1.55) |
|  |  |  |  |
| ***ARI2_DORFIN*** |  |  |  |
| *G. gallus/C. elegans* | 140 | 3-142 | 0.14±0.20 (1.13) |
|  |  |  |  |
| ***ARI2_ARA54*** |  |  |  |
| *G. gallus/C. elegans* | 137 | 5-141 | 0.19±0.20 (1.25) |
|  |  |  |  |
| ***ARI2_TRIAD3*** |  |  |  |
| *G. gallus/D. rerio* | 77 | 1-77 | -0.39±0.20 (0.17) |
| *G. gallus/D. rerio* | 62 | 79-140 | 0.39±0.15(0.08) |
| *M. musculus/D. rerio* | 46 | 2-47 | -0.37±0.19 (1.09) |
| *M. musculus/D. rerio* | 66 | 68-133 | 0.36±0.15(0.63) |
| *H. sapiens/D. rerio* | 92 | 50-141 | 0.23±0.12 (2.33) |
| *H. sapiens/D. rerio* | 91 | 48-138 | 0.23±0.12 (2.40) |
| *H. sapiens/D. rerio* | 91 | 49-139 | 0.23±0.12 (2.40) |
|  |  |  |  |
| ***ANKIB1_PAUL*** |  |  |  |
| *M. musculus/D. rerio* | 18 | 3-20 | -1.61±0.49 (2.13) |
| *M. musculus/D. rerio* | 103 | 22-124 | 0.23±0.21 (2.48) |
| *H. sapiens/D. rerio* | 110 | 22-131 | 0.20±0.20 (2.00) |
|  |  |  |  |
| ***ANKIB1_PARKIN*** |  |  |  |
| *H. sapiens/G. gallus* | 53 | 88-140 | -0.36±0.18 (1.85) |
| *H. sapiens/G. gallus* | 60 | 23-82 | 0.18±0.09 (2.48) |
|  |  |  |  |
| ***ANKIB1_RNF144*** |  |  |  |
| *G. gallus/D. rerio* | 21 | 76-96 | -1.14±0.59 (0.86) |
| *G. gallus/D. rerio* | 7 | 99-105 | 1.71±0.75 (2.43) |
| *M. musculus/D. rerio* | 24 | 76-99 | -1.08±0.53 (1.37) |
| *H. sapiens/D. rerio* | 30 | 70-99 | -0.97±0.43 (0.47) |
|  |  |  |  |
| ***ANKIB1_IBRDC2*** |  |  |  |
| *G. gallus/D. rerio* | 35 | 46-80 | -0.97±0.41 (1.47) |
| *M. musculus/D. rerio* | 5 | 76-80 | -3.80±1.91 (1.86) |
| *M. musculus/D. rerio* | 4 | 102-105 | 3.75±1.11 (1.84) |
| *H. sapiens/D. rerio* | 40 | 62-101 | -0.85±0.38 (1.09) |
| *H. sapiens/D. rerio* | 4 | 102-105 | 3.75±1.11 (1.88) |
| *H. sapiens/M. musculus* | 17 | 60-76 | -0.47±0.17 (0.04) |
|  |  |  |  |
| ***ANKIB1_TRIAD3*** |  |  |  |
| *G. gallus/D. rerio* | 115 | 19-133 | 0.10±0.11 (1.44) |
| *M. musculus/D. rerio* | 77 | 19-95 | 0.23±0.14 (1.42) |
| *M. musculus/D. rerio* | 64 | 33-96 | 0.28±0.16 (2.45) |
| *M. musculus/D. rerio* | 64 | 34-97 | 0.28±0.16 (2.45) |
| *M. musculus/D. rerio* | 64 | 35-98 | 0.28±0.16 (2.45) |
| *M. musculus/D. rerio* | 96 | 38-133 | 0.15±0.13 (2.28) |
| *H. sapiens/D. rerio* | 77 | 19-95 | 0.23±0.14 (1.37) |
| *H. sapiens/D. rerio* | 64 | 33-96 | 0.28±0.16 (2.42) |
| *H. sapiens/D. rerio* | 64 | 34-97 | 0.28±0.16 (2.42) |
| *H. sapiens/D. rerio* | 64 | 35-98 | 0.28±0.16 (2.42) |
| *H. sapiens/D. rerio* | 96 | 38-133 | 0.15±0.13 (2.28) |
|  |  |  |  |
| ***PARC_PARKIN*** |  |  |  |
| *M. musculus/G. gallus* | 26 | 88-113 | -1.08±0.46 (1.17) |
| *H. sapiens/G. gallus* | 18 | 96-113 | -1.33±0.63 (1.26) |
|  |  |  |  |
| ***PARC_XAP3*** |  |  |  |
| *M. musculus/C.intestinalis* | 10 | 38-47 | 2.90±1.02 (2.43) |
| *H. sapiens/C. intestinalis* | 8 | 40-47 | 3.50±1.27 (2.16) |
|  |  |  |  |
| ***PARC_IBRDC2*** |  |  |  |
| *M. musculus/G. gallus* | 131 | 8-138 | -0.22±0.09 (2.41) |
| *M. musculus/G. gallus* | 131 | 9-139 | -0.22±0.09 (2.41) |
| *M. musculus/G. gallus* | 131 | 10-140 | -0.22±0.09 (2.41) |
|  |  |  |  |
| ***PARC_ARA54*** |  |  |  |
| *G. gallus/C. intestinalis* | 40 | 37-76 | 1.12±0.40 (0.90) |
| *H. sapiens/M. musculus* | 15 | 78-92 | -0.93±0.61 (0.82) |
|  |  |  |  |
| ***PAUL_IBRDC1*** |  |  |  |
| *M. musculus/D. rerio* | 3 | 6-8 | -6.67±1.20 (2.25) |
| *H. sapiens/D. rerio* | 18 | 5-22 | -2.33±0.78 (2.10) |
|  |  |  |  |
| ***PAUL_XAP3*** |  |  |  |
| *M. musculus/C.intestinalis* | 9 | 74-82 | 2.78±1.22 (2.43) |
| *H. sapiens/C. intestinalis* | 9 | 74-82 | 2.78±1.22 (1.41) |
| *M. musculus/D. rerio (a)* | 25 | 25-49 | 1.36±0.50 (1.95) |
| *H. sapiens/D. rerio (a)* | 13 | 37-49 | 1.92±0.78 (2.47) |
| *M. musculus/D. rerio (b)* | 30 | 23-52 | 1.43±0.50 (0.49) |
| *H. sapiens/D. rerio (b)* | 30 | 23-52 | 1.23±0.48 (1.19) |
|  |  |  |  |
| ***PAUL_IBRDC2*** |  |  |  |
| *D. rerio/C. intestinalis* | 3 | 47-49 | -6.67±2.40 (2.38) |
|  |  |  |  |
| ***PAUL_TRIAD3*** |  |  |  |
| *M. musculus/D. rerio* | 114 | 23-136 | 0.27±0.18 (1.58) |
| *H. sapiens/D. rerio* | 114 | 23-136 | 0.25±0.18 (0.72) |
|  |  |  |  |
| ***IBRDC1_RNF144*** |  |  |  |
| *M. musculus/D. rerio* | 17 | 25-41 | 0.65±0.27 (1.56) |
|  |  |  |  |
| ***IBRDC1_DORFIN*** |  |  |  |
| *M. musculus/D. rerio* | 14 | 21-34 | 0.93±0.53 (2.05) |
| *H. sapiens/D. rerio* | 14 | 21-34 | 1.00±0.52 (0.73) |
|  |  |  |  |
| ***PARKIN_RNF144*** |  |  |  |
| *H. sapiens/G. gallus* | 39 | 78-116 | 0.26±0.12 (2.49) |
| *H. sapiens/M. musculus* | 16 | 88-103 | 0.44±0.22 (1.32) |
|  |  |  |  |
| ***PARKIN_IBRDC2*** |  |  |  |
| *H. sapiens/G. gallus* | 10 | 96-105 | 1.50±0.64 (2.30) |
|  |  |  |  |
| ***PARKIN_DORFIN*** |  |  |  |
| *M. musculus/C. elegans* | 40 | 74-113 | 1.67±0.49 (1.08) |
| *H. sapiens/C. elegans* | 57 | 80-136 | 1.25±0.41 (2.30) |
| *H. sapiens/C. elegans* | 40 | 74-113 | 1.57±0.47 (2.08) |
|  |  |  |  |
| ***PARKIN_IBRDC3*** |  |  |  |
| *G. gallus/C. elegans* | 29 | 3-31 | -1.14±0.47 (2.27) |
| *G. gallus/C. elegans* | 103 | 34-136 | 0.69±0.28 (0.60) |
| *M. musculus/C. elegans* | 40 | 74-113 | 1.42±0.45 (2.10) |
|  |  |  |  |
| ***PARKIN_ARA54*** |  |  |  |
| *H. sapiens/G. gallus* | 2 | 45-46 | -7.00±1.00 (1.39) |
|  |  |  |  |
| ***XAP3_RNF144*** |  |  |  |
| *D. rerio (a)/C. intestinalis* | 7 | 46-52 | -3.43±1.54 (1.81) |
| *M. musculus/D. rerio (a)* | 105 | 25-129 | 0.13±0.14 (1.34) |
| *H. sapiens/D. rerio (a)* | 86 | 25-110 | 0.24±0.17 (1.58) |
|  |  |  |  |
| ***XAP3_IBRDC3*** |  |  |  |
| *M. musculus/D. rerio (a)* | 45 | 2-46 | 1.02±0.37 (1.76) |
| *M. musculus/D. rerio (a)* | 30 | 47-76 | -0.93±0.26 (0.03) |
| *H. sapiens/D. rerio (a)* | 45 | 2-46 | 1.00±0.37 (1.95) |
| *H. sapiens/D. rerio (a)* | 30 | 47-76 | -0.9±0.25 (0.05) |
|  |  |  |  |
| ***XAP3_TRIAD3*** |  |  |  |
| *M. musculus/D. rerio (a)* | 29 | 34-62 | -1.41±0.46 (0.38) |
| *H. sapiens/D. rerio (a)* | 29 | 34-62 | -1.41±0.47 (0.33) |
| *H. sapiens/M. musculus* | 2 | 86-87 | -2.50±0.50 (1.40) |
|  |  |  |  |
| ***RNF144_IBRDC3*** |  |  |  |
| *H. sapiens/M. musculus* | 24 | 80-103 | 0.17±0.08 (1.38) |
|  |  |  |  |
| ***RNF144_ARA54*** |  |  |  |
| *M. musculus/G. gallus* | 51 | 92-142 | -0.37±0.14 (2.07) |
| *M. musculus/G. gallus* | 81 | 7-87 | 0.11±0.09 (2.45) |
| *M. musculus/G. gallus* | 81 | 8-88 | 0.11±0.09 (2.45) |
| *M. musculus/G. gallus* | 81 | 9-89 | 0.11±0.09 (2.45) |
| *M. musculus/G. gallus* | 81 | 10-90 | 0.11±0.09 (2.45) |
| *H. sapiens/G. gallus* | 2 | 46-47 | 3.50±0.50 (1.45) |
| *H. sapiens/M. musculus* | 9 | 78-86 | -1.00±0.50 (0.94) |
|  |  |  |  |
| ***IBRDC2_DORFIN*** |  |  |  |
| *H. sapiens/M. musculus* | 30 | 46-75 | 0.13±0.06 (1.35) |
|  |  |  |  |
| ***IBRDC2_IBRDC3*** |  |  |  |
| *H. sapiens/G. gallus* | 36 | 22-57 | 0.47±0.15 (2.2) |
|  |  |  |  |
| ***IBRDC2_ARA54*** |  |  |  |
| *H. sapiens/G. gallus* | 16 | 62-77 | -1.12±0.63 (1.88) |
|  |  |  |  |
| ***IBRDC2_TRIAD3*** |  |  |  |
| *G. gallus/D. rerio* | 5 | 101-105 | -2.60±0.75 (1.89) |
| *M. musculus/D. rerio* | 7 | 101-107 | -2.29±0.68 (1.32) |
| *H. sapiens/D. rerio* | 7 | 101-107 | -2.14±0.63 (1.56) |
| *H. sapiens/M. musculus* | 2 | 75-76 | -1.50±0.50 (1.43) |
|  |  |  |  |
| ***DORFIN_IBRDC3*** |  |  |  |
| *G. gallus/D. rerio* | 9 | 42-50 | -2.33±0.91 (1.02) |
| *M. musculus/G. gallus* | 42 | 16-57 | 0.98±0.41 (0.54) |
| *H. sapiens/G. gallus* | 42 | 16-57 | 0.98±0.41 (0.56) |
|  |  |  |  |
| ***IBRDC3_ARA54*** |  |  |  |
| *M. musculus/C. elegans* | 93 | 50-142 | -0.62±0.29 (2.28) |
|  |  |  |  |
| ***IBRDC3_TRIAD3*** |  |  |  |
| *G. gallus/D. rerio* | 25 | 77-101 | 1.12±0.38 (2.10) |
